# Study of the effect of dryness and storage on *Ceratonia siliqua* L. stem extracts and evaluation of their insecticidal activity

**DOI:** 10.1038/s41598-025-93181-4

**Published:** 2025-04-01

**Authors:** Esraa A. Elhawary, Mohammed E. Gad, Maysa M. Hegazy, Reham M. Mostafa, Hattan S. Gattan, Mohammed H. Alruhaili, Abdelfattah M. Selim, Abadi M. Mashlawi, Abeer Mousa Alkhaibari, Saeed M. Alasmari, Mohamed M. Baz

**Affiliations:** 1 Department of Pharmacognosy, Faculty of Pharmacy, Ain-Shams University, Cairo, 11566 Egypt; 2 Department of Zoology and Entomology, Faculty of Science, Al-Azhar University, Nasr City, 11884 Cairo Egypt; 3 Biology Department, Faculty of Science, Jazan University, Jazan, Saudi Arabia; 4 Botany and Microbiology Department, Faculty of Science, Benha University, Benha, 13518 Egypt; 5 Department of Medical Laboratory Sciences, Faculty of Applied Medical Sciences, King Abdulaziz University, Jeddah, Saudi Arabia; 6 Department of Clinical Microbiology and Immunology, Faculty of Medicine, King AbdulAziz University, Jeddah, Saudi Arabia; 7 Special Infectious Agents Unit, King Fahad Medical Research Center, King AbdulAziz University, Jeddah, Saudi Arabia; 8 Department of Animal Medicine (Infectious Diseases), College of Veterinary Medicine, Benha University, Toukh, 13736 Egypt; 9 Department of Biology, College of Science, Jazan University, 45142 Jazan, Kingdom of Saudi Arabia; 10 Department of Biology, Faculty of Science, University of Tabuk, 71491 Tabuk, Saudi Arabia; 11 Department of Biology, Faculty of Science and Arts, Najran University, 1988 Najran, Saudi Arabia; 12 Department of Biology, Faculty of Education and Arts, Sohar University, Sohar, 311 Oman; 13 Entomology Department, Faculty of Science, Benha University, Benha 13518, Egypt

**Keywords:** *Ceratonia siliqua*, Solanaceae, Biocontrol, Larvicidal activity, *Culex*, *Musca*, Plant sciences, Parasitology

## Abstract

Vector-borne diseases continue to transmit many dangerous pathogens to humans. After decades of continuous use of insecticides, many types of vectors have shown the ability to build resistance to them. This has necessitated the development of more efficient and environmentally friendly alternatives in the form of bioinsecticides. Plants contain a wide range of phytochemicals with specific targeting, rapid biodegradability, environmental sustainability and a variety of medicinal properties, making them a valuable source of biologicals. Moreover, this has led to the development of highly effective new drugs. This study aimed to identify the active ingredients in *Ceratonia siliqua* L., gathered from two consecutive fruiting seasons which were then divided into *C. siliqua* fresh (CSF), dry (CSd), and old (stored) stem (CSO) extracts *Ceratonia siliqua*. Metabolomics profiling was performed using UPLC/MS and multivariate data analysis. The UPLC/MS study resulted in the tentative identification of 54 secondary metabolites. These compounds included flavonoids, phenolic acids, withanolides, terpenoids, phenylpropanoids, etc. CSd showed the highest number of identified components followed by CSO and CSF. The % identification was nearly equal in the negative ion mode for the three extracts while for the positive ion mode it followed the order of CSF > CSd > CSO. After several exposure periods, the plant methanol extracts in this research showed significant insecticidal activity against mosquito larvae, *Cx. pipiens*, and housefly larvae *M. domestica*. (CSd) demonstrated the highest insecticidal activity (100 MO%) against *Cx. pipiens* (LC_50_ = 0.09 and 0.07 mg/ml) following 24- and 48-hour post-treatments at 1.0 mg/ml. The (CSF) was the most effective on *M. domestica* larvae (LC_50_ = 2.32 and 1.80 mg/ml), 24 and 48 h post-treatment with 25 mg/ml concentration. Both CSd and CSF extracts were the most effective at killing mosquito and house fly larvae, followed by the CSO extract. Therefore, *C. siliqua* extracts may serve as an effective bio-agent for specific vector-borne infection control.

## Introduction

With over 100 identified cultivars, *Ceratonia siliqua* L. (Carob, St John’s‑bread or locust bean) is an evergreen shrub or tree that is native to the Mediterranean basin and is extensively grown in regions with warm temperate and subtropical climates. Carob may thrive in a variety of soil types, including dry, very calcareous, and nutrient-poor soils. Although carob is primarily cultivated by females, it is dioecious or occasionally hermaphrodite. Insects are mostly responsible for pollination, whereas mammals predominantly distribute the comparatively big seeds through fruit consumption. A stiff seed coat forces seeds into dormancy, and seeds that have undergone natural or artificial scarification germinate easily. Extremely drought-tolerant, it avoids drought by using water sparingly. Additionally, carob is highly resistant to fire and salt, and it can help prevent wildfires from spreading by creating fuel discontinuity^1^.

The carob tree is indigenous to the Mediterranean region, which includes the Canary Islands, Macaronesia, the Levant, the Middle East of Western Asia, Iran, Southern Europe, Northern Africa, and the larger Mediterranean islands. Indirectly derived from the Greek word for a carob seed, kerátion, the carat is a unit of mass for gemstones and purity for gold. The only Mediterranean tree that blooms primarily in the fall (September–November) is *C. siliqua*. However, like other fruit and nut trees, the duration and timing of the flowering period are dependent on the local climate. The size of carob beans varies greatly and is impacted by a variety of environmental conditions, including fruit set and pollination level. Insects, particularly bees, flies, wasps, and night-flying moths, are responsible for dispersing pollen. Feed for both ruminants and non-ruminants is provided by *C. siliqua* pods. The seed’s embryo and endosperm can be crushed and fed to pets. Nowadays, Mediterranean nations that practice zero-grazing use the fodder. Condensed tannins make up 16–20% of the dry weight of ripe carob pods. The pulp’s tannins have anti-diarrheic properties. Pharmaceutical goods are prepared using ground pulp and seed endosperm^2,3^.

Carob trees are used to make sugar-rich animal feed and for human use. Currently, nevertheless, the primary focus is on producing seeds for the extraction of gum from the endosperm, which is utilized as a stabilizer in a variety of commercial food products. The carob tree is ideal for part-time farming because of its exceptional qualities, such as its rusticity and tolerance to drought, as well as the fact that it produces with less orchard management. The primary drawback of carob is its susceptibility to cold. Furthermore, compared to traditional carob plots, modern carob orchards begin producing four years after budding and they gradually increase production in response to deficiency watering and minimal cultural care^4^.

Although this crop has not gotten much attention up to this point, it is now being reemphasized as a viable option for coastal agriculture diversification and revitalization in dryland (500 mm) or supplemental drip irrigation locations with a Mediterranean climate^1^.

Flavonols including quercetin, myricetin, kaempferol and their glucosidic derivatives are especially abundant in carob fruits. The most prevalent flavonoids in carob are often quercetin and myricetin rhamnosides. Flavonones (naringenin), isoflavones (genistein) and flavones (apigenin, luteolin and chrysoeriol) are not very abundant. The most distinctive class of polyphenols found in carob fruits are tannins, which also give them their astringent flavor. The tannin content in carob juice is 10 times greater than that of grape juice and it decreases as the fruit ripens^5^. Numerous researches have demonstrated the biological activity of carob, including antibacterial, anticancer and antioxidant properties^6^.

In addition to its many other benefits, using the seeds or leaves of the carob plant to repel pests is applicable, particularly those that spread disease. Worldwide, vector-borne illnesses continue to be a major public health concern, particularly in tropical and subtropical regions. Over three billion people live in unhealthy environments, further endangering public health. Arthropod vectors have the ability to transmit a wide range of harmful pathogens, which can result in the spread of infectious diseases that can harm both humans and animals^7,8^. Under certain conditions, many diseases can spread directly from person to person. These conditions include interactions between viruses, hosts, vectors, susceptible populations and the presence of disease reservoirs^9^.

Mosquito-transmitted diseases such as filaria, dengue and malaria have long documented numerous fatalities from mosquito-transmitted illnesses. According to a recent study, 88 (2.5%) of the 3578 species of mosquitoes are carriers for 78 different human diseases^10^. Furthermore, researchers believe that 243 mosquito species, accounting for 6.8%, could potentially transmit human diseases. Nonetheless, the chance of mosquito-borne viruses (MBVs) spreading to nations that may not have a history of MBVs is growing due to the sharp increase in worldwide travel. Female mosquitoes feed on vertebrate blood to get the necessary nutrients for laying eggs, which spreads hundreds of virus particles that may be present in their saliva^11^.

The house fly is wide distribution and can mechanically carry a variety of diseases to humans^12^. This is because adult houseflies have a keen sense of smell and feed on things like human food, animal excrement, perspiration, trash and moist or decaying material from pet waste^12^. Also, its vomit or excrement can carry viruses, helminthic protozoa and bacteria including *E. coli*, *Shigella* species and *Salmonella*, among almost a hundred diseases that can infect humans and animals.

Various classes of synthetic insecticides have been widely used to control disease vectors, including pyrethroid insecticides and organophosphate insecticides, to control mosquitoes and houseflies^13^. However, despite these insecticides’ ability to reduce the invasiveness of disease vectors, particularly during disease outbreaks or increased pest density, they also pollute the environment, endanger human health, harm non-target animals and, most importantly, increase the resistance of these insects to the insecticides used^14^.

As a result, it was necessary to find a suitable alternative agent to synthetic pesticides, which represent bioinsecticides. Biopesticides have enormous potential and provide a more cost-effective, accessible and environmentally safe alternative to conventional insecticides; botanicals have attracted much attention in many medical and industrial fields^15^. These alternative techniques can be a powerful tool in integrated pest management plans, delaying the development of resistance to conventional insecticides^16^. Several studies have documented the insecticidal properties of plant extracts and essential oils against houseflies^17^. Limonene, myrcene, terpineol, linalool and pulegone are some of the monoterpenoids that can kill houseflies. Therefore, plant extracts or essential oils can replace synthetic insecticides to eliminate houseflies and other harmful insects^18^ .

Thus, this article aimed at exploring the effect of drying and storage on the metabolite composition of *Ceratonia siliqua* L. (Carob) stem extracts followed by investigation of the extract’s activity against different insects, with medical importance, such as *Culex pipiens* and *Musca domestica*.

## Materials and methods

### Plant collection

*Ceratonia siliqua* L. stems were collected during two consecutive seasons August and September of (2022–2023) (2023–2024) from Mazhar Botanic Garden, 26th of July Corridor, Nahia, Imbaba, Giza Governorate 3,648,030, Giza Governorate 12,511 (30.066451667289048, 31.14399558465528). The collected plant was then identified and authenticated by Dr. Trease Labib, a plant taxonomy consultant at the Egyptian Ministry of Agriculture and a voucher specimen was deposited at the herbarium of the Pharmacognosy Department, Faculty of Pharmacy, Ain Shams University under the code: PHG-P-CS-508.

### Extract preparation

The collected fresh, dried and stored stems were separately cut down to suitable size. About (1Kg) from each stem group was soaked separately in 70% methanol then filtered and the excess solvent was evaporated using Rotavap^®^. The extraction process was repeated three times. The resulting extracts were weighed and recorded as: old or stored for stems collected during season 2022–2023 (CSO), fresh for stems collected during season 2023–2024 (CSF) and dried for stems collected and dried during season 2023–2024 (CSd) and their weights were: old methanol extract (CSO, 15 g), Fresh methanol extract (CSF, 12 g) and dried methanol extract (CSd, 15 g). The prepared extracts were dried till no residual solvent and kept in freezer for further use.

### UPLC/MS analysis

The UPLC/ESI/MS analysis was executed for the three *Ceratonia siliqua* L. stem extracts adopting the method of^19–24^. UPLC/ESI/MS in both positive and negative ion acquisition modes were carried out on a XEVO TQD triple quadrupole instrument, Waters Corporation, Milford, MA01757 U.S.A, mass spectrometer. Chromatographic separation of the sample was done by injecting 10 µl into UPLC instrument equipped with reverse phase C-18 column (ACQUITY UPLC - BEH, 2.1 × 50 mm column; 1.7 μm particle size). The sample (100 µg/mL) solution was prepared using HPLC grade methanol, filtered using a membrane disc filter (0.2 μm) disc and degassed by sonication before injection then subjected to LC/ESI/MS analysis. The gradient mobile phase comprises two eluents: eluent A is H_2_O acidified with 0.1% formic acid and eluent B is MeOH acidified with 0.1% formic acid. Elution was made at flow rate 0.2 mL/min as follows: (10%B) from 0 to 5 min.; (30% B) from 5 to 15 min.; (70% B) from 15 to 22 min.; (90% B) from 22 to 25 min. and (100% B) 25–29 min. The analysis was accomplished using negative ion mode as follows: source temperature 150 °C, cone voltage 30 eV, capillary voltage 3 kV, desolvation temperature 440 °C, cone gas flow 50 L/h, and desolvation gas flow 900 L/h. Mass spectra were recorded in Electrospray ionization (ESI) (negative and positive ion modes) (*m/z* 100–1000). UPLC/MS data were processed using Masslynx 4.1 software and tentative identification was done by comparing their retention times (Rt), mass spectra and fragmentation patterns with reported data.

### Larvicidal assay

#### Mosquito colony

For all tests, *Cx. pipiens* mosquito larvae were obtained from the Entomology Department, Faculty of Science, Benha University, Egypt. The larvae were kept in a laboratory with controlled conditions (27 ± 2 °C, 75–80% relative humidity and a 12:12 h light/dark photoperiod). The gathered larvae were reared in an enamel plate measuring 25 × 20 × 10 cm, containing 2 L of dechlorinated water and were nourished with fish food (Tetramin^®^) and pulverized dog biscuits 1:3. Adults were supplied with an 8–10% sucrose solution as a nutritional resource. Both adults and larvae were sustained in identical laboratory circumstances^25^.

#### Housefly colony

Adult house flies were captured from the Benha vegetable markets in Qalyubia, Egypt. Subsequently, we housed them in wooden cages of 30 × 30 × 30 cm³ with wire covers and maintained them at room temperature (30–32 °C) in the Insect Breeding Laboratory, Division of Entomology and Environment, Department of Entomology, Faculty of Science, Benha University. Cotton wool was wetted with food, which was a mixture of 10% syrup and 10% milk. We cooked 300 g of mackerel in a plastic tray with dimensions of 18 × 25 × 9 cm2 with a combination of desiccated bread and ragweed, establishing an optimal habitat for house flies’ larvae to nourish themselves and deposit their eggs. The inquiry encompassed recently emerging adults^26^.

#### Larvicidal bioassays

*C. siliqua* plant extracts were evaluated in accordance with WHO^27^ to determine their efficacy in controlling the third larval instar of *Cx. pipiens*. Twenty-five larvae populated a glass beaker containing 250 ml of various concentrations (0.01, 0.025, 0.05, 0.1, 0.2, 0.5 and 1.0 mg/ml). Both the experimental and control groups were administered just with water solvent. The experiments were performed three times. The death rates of *Cx. pipiens* larvae were documented 24- and 48-hours post-treatment (PT) at 27 ± 2 °C and 80% relative humidity (RH).

*Musca domestica* bioassays were conducted to assess the impact of plant extracts on fly larvae by the feeding method, wherein the larvae were placed in a treated culture medium. Fifteen early third-instar larvae were placed in little paper cups 5 cm in diameter and 7 cm in height, containing 5 g of rearing medium. The cups were subsequently subjected to treatment with 0.5, 1.0, 0.5, 5.0, 10, and 25 mg/ml plant methanol extracts. The treated and untreated cups were covered with a cotton cloth secured by a rubber band to inhibit larvae from escaping. After 24 and 48 h, dead larvae were enumerated and subsequently, 5 g of sawdust were introduced into each cup for pupation. The experiment was conducted three times.

### Statistical analysis

The data were analyzed by the software, SPSS V23 (IBM, USA), for doing the Probit analyses to calculate the lethal concentration (LC) values and the one-way analysis of variance (ANOVA) (Post Hoc/Turkey’s HSD test). The significant levels were set at *P* < 0.05.

## Results

### UPLC/MS analysis of three extracts from *Cetratonia siliqua* stem

The stem of *Ceratonia siliqua* L. (*C. siliqua*) commonly known as Carob can be regarded as a waste since the fruits are the most common edible part of the plant. The stem was extracted through different seasons and also the dried and fresh stem extracts were prepared and compared. Three stem extracts were annotated as the fresh stem extract (CSF), the dried stem extract (CSd) and the old or stored stem extract (CSO) (stored for one season). The three extracts were analyzed using UPLC/MS in positive and negative ion modes. Moreover, the three extracts were subjected to a comparative, qualitative and quantitative assessment of their metabolite contents (Table 1). The classes of identified 2^ry^ metabolites fall in the categories presentedFig. gs. 1 & 2. Flavonoids constituted nearly 30% of the identified components followed by phenolic acids, tannins, fatty acids and otheFig. ig. 2). A comparison between the BPI chromatograms for the three extracts, in both positive and negative ion modes, was illustratedFig. gs. 3 and 4.

**Table 1:** The tentatively identified components from the stem extracts of *Ceratonia siliqua* through UPLC/MS.

No.	Component	Molecular formula	Chemical class	*R*_t_ (min.)	[M-H]^−^ m/z	[M + H]^+^ m/z	Different stem extracts of Ceratonia siliqua (area between brackets for + ve mode)			Reference(s)
							CSF	CSd	CSO	
1	Quinic acid derivative	–	Phenolic acid	0.79	381	–	0.82	**14.57**	–	^86^
2	Galloyl-*O*-hexoside	C_13_H_16_O_10_	Tannin	0.94	331	–	–	**9.10**	**12.23**	^50^
3	Tricetin dimethyl ether deriv.	–	Flavonoid	1.08	–	331	–	**10.05**	**16.21**	^28^
4	*di*-Galloyl hexose^*^	C_20_H_20_O_14_	Tannin	1.18	483	485	2.59	**13.43** (0.40)	**17.69** (2.96)	^20^
5	Apigenin‑*O*‑hexouronide^*^	C_21_H_20_O_10_	Flavonoid	1.76	443	–	–	–	0.26	^28^
6	(+) Epicatechin-*O*- hexoside-hexoside	C_27_H_36_O_17_	Tannin	2.15	613	–	0.41	**4.98**	**13.39**	^49^
7	Feruloyl-quinic acid	C_17_H_20_O_9_	Phenolic acid	2.53	367	–	**3.01**	**8.34**	1.97	^87^
8	Chrysoeriol-*O*-hexournide	C_22_H_22_O_11_	Flavonoid	2.67	475	–	–	2.74	–	^37^
9	*tri-*Galloyl-hexose^*^	C_27_H_24_O_18_	Tannin	2.86	635	–	0.94	–	–	^51^
10	3-[(2E)-3,7-dimethylocta-2,6- dienyl]-2,4-dihydroxy-6-[(E)-2- phenylethenyl] benzoic acid	C_25_H_28_O_4_	Stilbene	3.07	391	–	–	0.35	–	^57^
11	*di*-Galloyl hexose derivative^*^	C_26_H_28_O_16_	Tannin	3.68	595	–	–	0.08	–	^52^
12	Quercetin-*di*-hexoside	C_27_H_30_O_17_	Flavonoid	5.06	627	651	0.40	1.97	1.50	^35^
13	*iso*-Schaftoside^*^	C_26_H_28_O_14_	Indole alkaloid	5.52	-	565	**11.10**	**8.29**	1.27	
14	Quercetin-*O*-hexoside^*^	C_21_H_19_O_12_	Flavonoid	6.08	463	465	1.45	2.46	2.31	^29–31^
15	*tetra*-Galloyl-hexose^*^	C_34_H_28_O_22_	Tannin	6.26	787	–	–	–	1.33	^51^
16	Quercetin-*O*-pentoside	C_21_H_20_O_11_	Flavonoid	6.65	447	449	0.36	2.47	2.96	^22,36^
17	Chlorogenic acid derivative	–	Phenolic acid	8.34	451	–	0.39	1.28	1.52	^44^
18	Apigenin^*^	C_15_H_10_O_5_	Flavonoid	8.65	269	–	–	0.23	–	^32^
19	Oxiranedioctanoic acid	C_18_H_32_O_5_	Fatty acid	8.90	327	–	–	0.74	0.55	^38^
20	Tricetin *di* methyl ether^*^	C_17_H_14_O_7_	Flavonoid	9.50	329	331	(11.90)	2.31 (8.44)	**3.22**	^33^
21	Resveratrol^*^	C_14_H_12_O_3_	Stilbene	9.82	–	229	–	**6.29**	–	^58^
22	Caffeic acid hexoside	C_15_H_18_O_9_	Phenolic acid	11.39	–	343	**12.48**	**6.29**	–	^19,24^
23	Caffeic acid derivative^*^	–	Phenolic acid	13.89	297	–	**20.92**	**10.15**	**10.36**	^45^
24	*di*-Hydroxyoctadecatrienoic acid^*^	–	Fatty acid	14.78	311	–	**32.51**	**11.51**	**4.03**	^33^
25	*di*-Hydrophilonotisflavone	C_30_H_19_O_12_	Flavonoid deriv.	15.21	571	631	–	–	1.40 (**12.50**)	^38^
26	Chlorogenic acid	C_16_H_18_O_9_	Phenolic acid	15.71	353	–	**3.40**	**6.76**	0.45	^19–24,42^
27	Cyanidin-pentoside	C_36_H_43_O_24_	Anthocyanin	15.77	–	421	11.62	–	–	^31^
28	Unknown diterpene^*^	–	Diterpenoid	16.01	325	–	**14.90**	**3.95**	1.74	^33^
29	9-Oxooctadecadienoic acid	C_18_H_30_O_3_	Fatty acid	16.48	293	–	**7.81**	2.38	–	^55^
30	Caffeoyl-2-hydroxyethane-1,1,2-tricarboxylic acid	–	Phenolic acid	17.76	339	–	2.75	1.28	–	^46,47^
31	Hydroxy-pentacosanoic acid^*^	–	Fatty acid	17.78	397	–	–	0.20	–	^33^
32	Paxanthone	C_19_H_16_O_6_	Miscellaneous	18.73	340	–	0.36	–	–	^55^
33	Naringenin^*^	C_15_H_12_O_5_	Flavonoid	19.51	271	–	–	0.32	**12.82**	^32^
34	Dimethyl kuraridin	C_28_H_36_O_6_	Prenylated flavonoid	19.76	425	–	0.09	0.09	–	^39,40^
35	Naringenin derivative^*^	–	Flavonoid	20.05	469	–	0.07	–	–	^34^
36	Luteolin hexouronide derivative	–	Flavonoid	20.41	513	–	0.08	–	–	^41^
37	Gnoderic acid B	C_30_H_44_O_7_	Triterpenoid	22.42	497	–	–	-	0.11	^53^
38	Ursolic acid methyl ester	C_31_H_50_O_3_	Triterpenoid	23.01	-	471	**17.34**	**5.48**	–	^24,54^
39	Isopentyl dihexose	–	Miscellaneous	25.56	–	413	**12.29**	**6.45**	**11.57**	
40	Crypto-chlorogenic acid	C_16_H_18_O_9_	Phenolic acid	28.31	355	–	–	–	0.16	^43^
41	*O*-Caffeoyl‐*O*‐succinyl‐*O*‐ [(hydroxyphenyl)-acetyl]‐ methyl quinate-*O*-caffeoyl‐*O*‐succinyl‐*O*‐ [(hydroxyphenyl)-acetyl]‐ methyl quinate	–	Phenolic acid	30.19	–	631	–	**5.16**	–	^48^
42	*tri*-Hydroxy-octadecenoyl-glycero-phosphate	–	Fatty acid	31.06	465	–	–	–	2.42	^56^
43	5,6,7-*tri*-Hydroxy-2,3- dihydrocyclopenta[b]chrom ene-1,9-dione-3-carboxylic acid hexoside	–	Miscellaneous	31.15	453	–	–	–	0.35	^88^
	% Identification (No. of compounds) Negative ion mode Positive ion mode						93.26% (18) 76.75% (6)	100.00% (23) 56.45% (8)	94.04% (23) 40.29% (3)	

*for the compounds previously identified from genus *Ceratonia* from literature, CSF: *Ceratonia siliqua* fresh stem extract, CSd: *Ceratonia siliqua* dried stem extract and CSO: *Ceratonia siliqua* old (stored) stem extract.

**Fig. 1:**
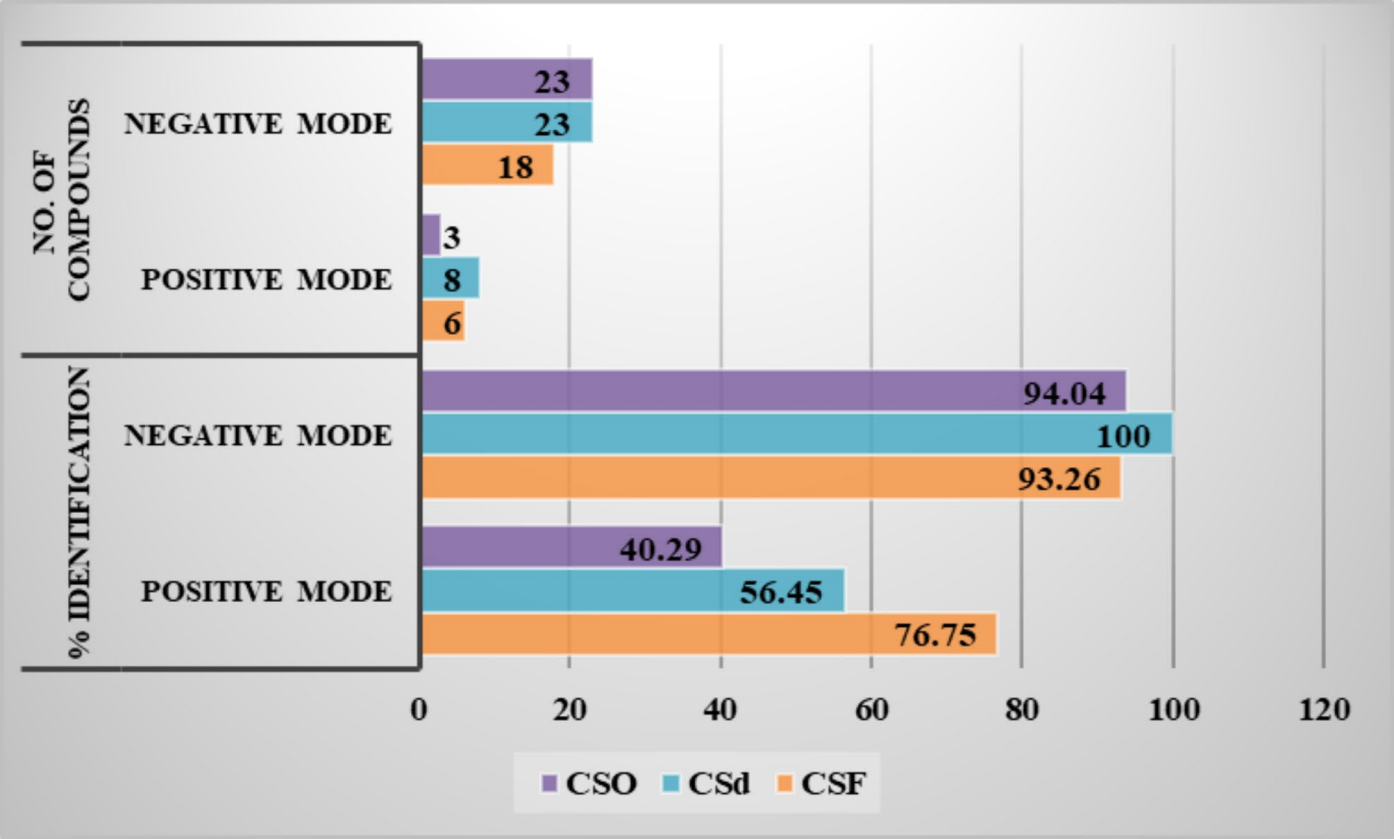
Bar chart showing the % identification and number of identified components/mode for the three *C. siliqua* stem extracts.

**Fig. 2:**
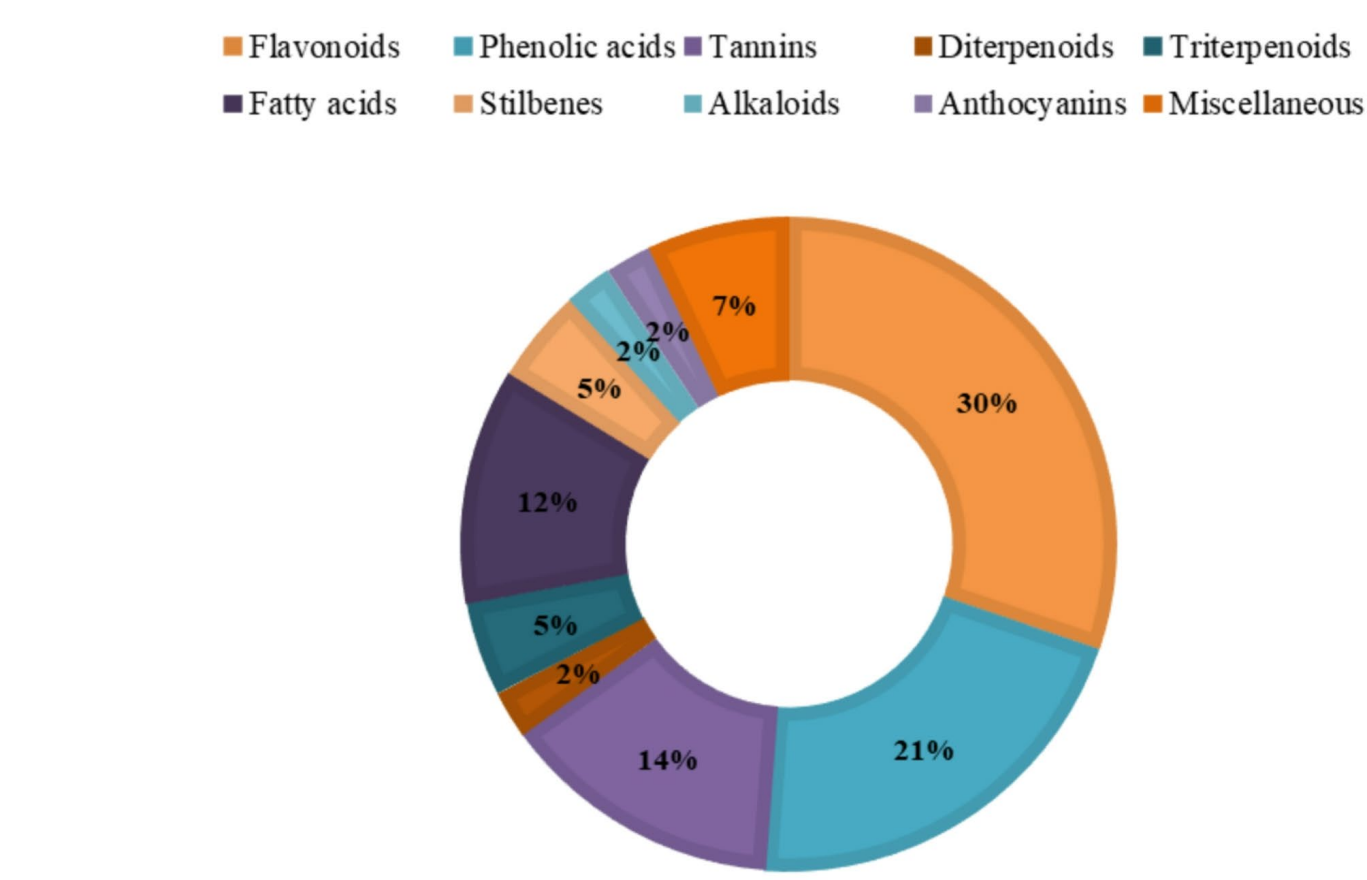
Sunburst showing the percentage of each 2^ry^ metabolite category tentatively identified from the three *C. siliqua* stem extracts.

**Fig. 3:**
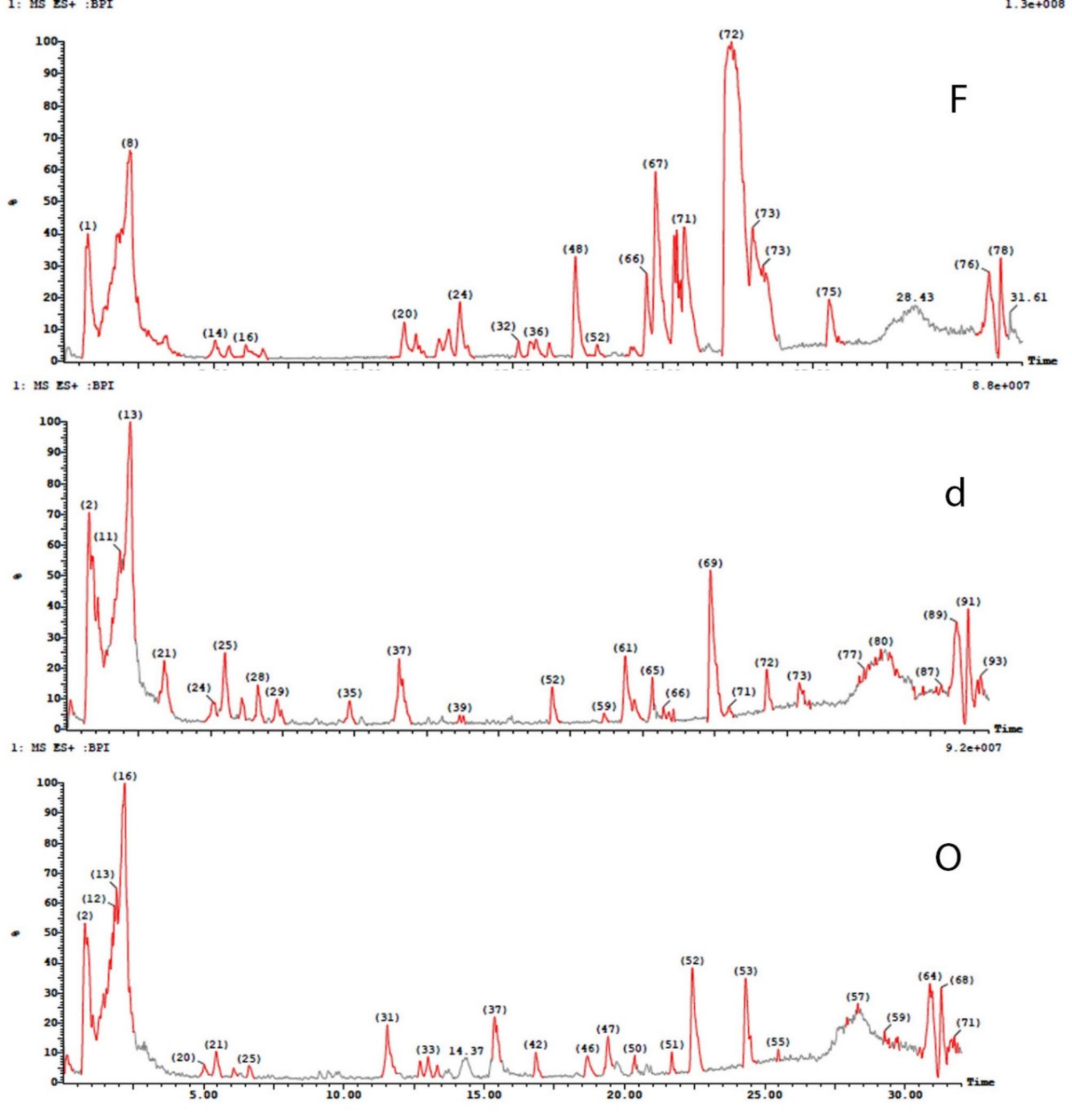
BPI chromatograms in positive ion mode for the three *C. siliqua* stem extracts (F: fresh, d: dried and O: old stem extracts).

**Fig. 4:**
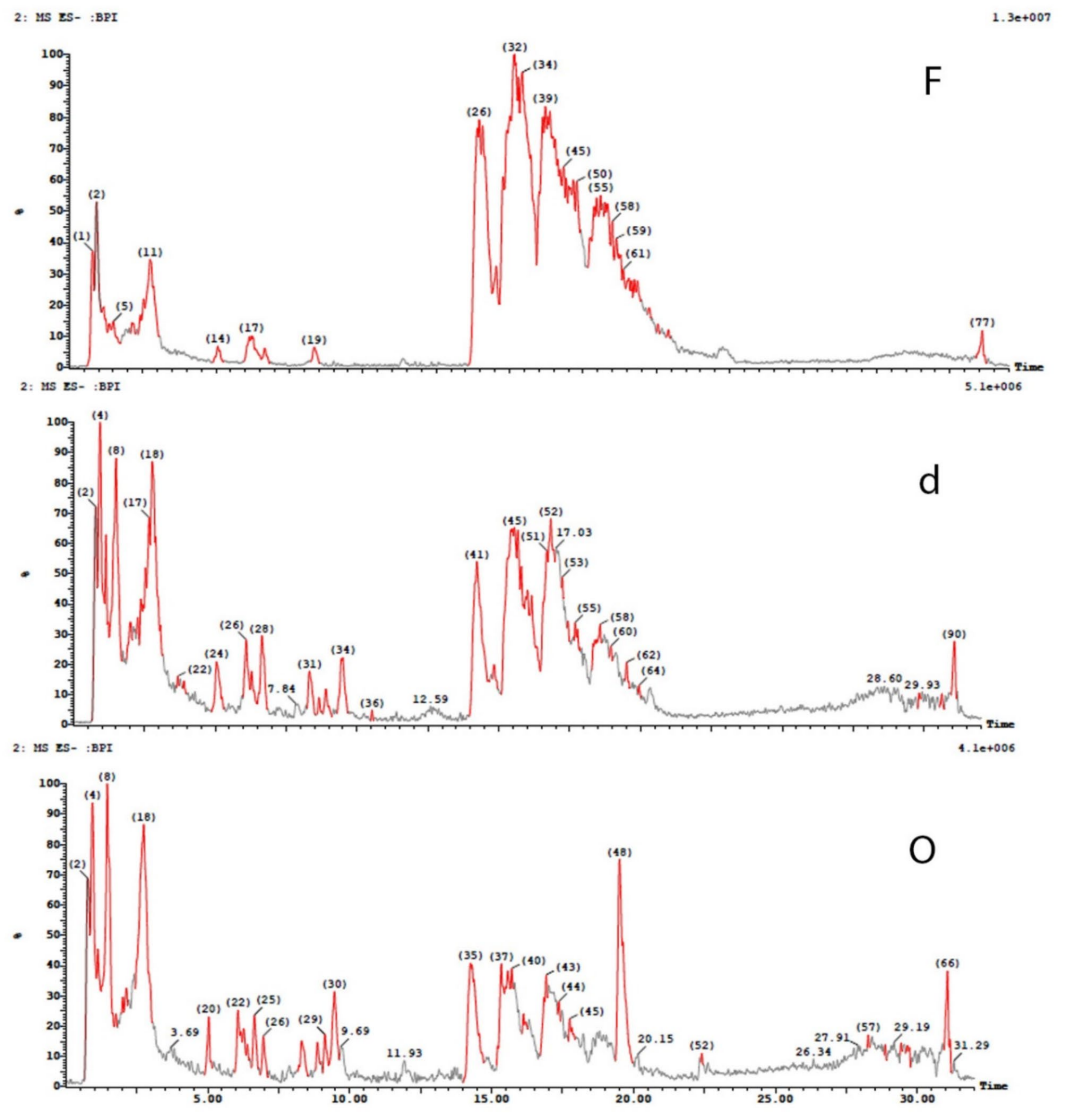
BPI chromatograms in negative ion mode for the three *C. siliqua* stem extracts (F: fresh, d: dried and O: old stem extracts).

### Flavonoids

Thirteen flavonoids and flavonoid derivatives were tentatively identified from the three *C. siliqua* stem extracts (Table 1). Six flavonoids were previously reported from genus *Ceratonia* and were annotated by (*) in Table 1. The aforementioned flavonoids were marked as compounds 5, 14, 18, 20, 33 and 35. They were tentatively defined as apigenin‑*O*‑hexouronide^28^, quercetin-*O*-hexoside^29–31^, apigenin^32^, tricetin *di* methyl ether^33^, naringenin (12.82% CSO)^32^ and naringenin derivative^34^, respectively. Compound 5 presented its peak at *m/z* 443 with molecular formula of C_21_H_20_O_10_ (only in CSO). In a similar fashion, compound 14 had a deprotonated peak at *m/z* 463(465) with molecular formula of C_21_H_19_O_12_. In addition to that, two other quercetin derivatives were tentatively found as compound 12 (quercetin-*di*-hexoside, *m/z* 627(651) showing a Na adduct in positive mode, C_27_H_30_O_17_)^35^ and compound 16 (quercetin-*O*-pentoside, *m/z* 447(449), C_21_H_20_O_11_)^22,36^. Similarly, other components were tentatively assigned as flavonoids and were presented in Table 1 as compounds 3, 8, 25, 34 and 36 which were defined as tricetin dimethyl ether deriv. (CSd and CSO only)^28^, chrysoeriol-*O*-hexournide (CSd only)^37^, *di*-hydrophilonotisflavone (CSO only)^38^, dimethyl kuraridin (CSF and CSd only, a prenylated flavonoid)^39,40^ and luteolin hexouronide derivative (CSF only)^41^, respectively (Table 1).

### Phenolic acids

Chlorogenic acid^19–24,42^ together with crypto-chlorogenic acid^43^ and a chlorogenic acid derivative^44^ were detected as compounds 26, 40 and 17, respectively where their deprotonated molecular ion peaks appeared at *m/z* 353, 355 and 451, respectively. Similarly, compounds 22, 23, 30 and 41 were found to be caffeic acid derivatives. Compound 22 presented its peak at *m/z* 343 (+ ve mode) with molecular formula of C_15_H_18_O_9_ (2.48% CSF and 6.29% Csd) and it was defined as caffeic acid hexoside^19,24^. Compound 23 showed a peak at *m/z* 297 and it was previously identified from genus *Ceratonia* (20.92% CSF, 10.15%, Csd, 10.36% CSO) and was traced as caffeic acid derivative^45^. Besides, caffeoyl-2-hydroxyethane-1,1,2-tricarboxylic acid^46,47^ and *O*-caffeoyl‐*O*‐succinyl‐*O*‐[(hydroxyphenyl)‐acetyl]‐methyl quinate-*O*‐caffeoyl‐*O*‐succinyl‐*O*‐ [(hydroxyphenyl)‐acetyl]‐methyl quinate^48^ were assigned to the deprotonated peaks appearing at m/z 339 and 631 (+ ve mode), respectively (Table 1).

### Tannins

Tannins presented the third most abundant class of components tentatively identified from the three *Ceratonia* extracts. A deprotonated peak was detected at [M-H]^-^ *m/z* 613 (4.985 CSd, 13.39% CSO) and it was assigned to (+) epicatechin-*O*- hexoside-hexoside^49^. The rest of the identified tannins belonged to the hydrolysable tannin class and all of them carried one or more galloyl moiety also they were previously isolated from genus *Ceratonia*. They were marked as compounds 2, 4, 9, 11 and 15 with deprotonated peaks at *m/z* 331, 483(485), 635, 595 and 787, respectively which lead to their tentative identification as galloyl-*O*-hexoside (9.10% CSd, 12.23% CSO)^50^, *di*-galloyl hexose (13.43% CSD, 17.69% CSO, 2.59% CSF)^20^, *tri-*galloyl-hexose (0.94% CSF only)^51^, *di*-galloyl hexose derivative (traces CSd only)^52^ and *tetra*-galloyl-hexose (1.33% CSO only)^51^, respectively (Table 1).

### Triterpenoids

Two peaks were detected at *m/z* 497 and 471 (+ ve mode) and were assigned to the triterpenoids gnoderic acid B (traces CSO only)^53^ and ursolic acid methyl ester (17.34% CSF and 5.48% CSd)^24,54^, respectively (Table 1).

### Fatty acids

Five fatty acids were detected from the stem extracts including: *di*-hydroxyoctadecatrienoic acid (*m/z* 311, appear as a major peak in the three extracts; 32.51% CSF, 11.51% CSd and 4.03% CSO)^33^ and hydroxy-pentacosanoic acid (*m/z* 397)^33^ which were previously reported from genus *Ceratonia* in addition to oxiranedioctanoic acid (*m/z* 327)^38^, 9-oxooctadecadienoic acid (*m/z* 293, 7.81% CSF, 2.38% CSd)^55^ and *tri*-hydroxy-octadecenoyl-glycero-phosphate (*m/z* 465, 2.42% CSO)^56^ as presented in Table 1.

### Stilbenes

Two stilbene peaks were traced at *m/z* 391 and *m/z* 229 (+ ve mode) which were tentatively assigned to 3-[(2E)-3,7-dimethylocta-2,6- dienyl]-2,4-dihydroxy-6-[(E)-2- phenylethenyl] benzoic acid^57^ and resveratrol (6.29% CSd only, previously reported from genus *Ceratonia*) (Table 1)^58^.

### Alkaloids

An indole alkaloid which was previously identified from genus *Ceratonia* was detected at *m/z* 565 (+ ve mode) with molecular formula of C_26_H_28_O_14_ (11.10% CSF, 8.29% CSd and 1.27% CSO) thus it was identified as *iso*-schaftoside (Table 1)^59^.

### Anthocyanins

One anthocyanin peak was detected at *m/z* 421 + ve ion mode (11.62% CSF only) and it was tentatively defined as cyanidin-pentoside (Table 1)^31^.

### Insecticide activity

#### Mosquito larvicidal activity

This research assessed the effects of *Ceratonia siliqua* L. stem extracts on the 3rd instar larvae of *Culex pipiens*. All examined plant extracts in this study exhibited significant insecticidal efficacy against mosquito larvae, *Cx. pipiens*, following various exposure time. It was found that *Cx. pipiens* larvae died at a rate of 100% after being treated with 1 mg/ml methanol extracts of *C. siliqua* dry (CSd) and fresh (CSF) and 96% for old (CSO) (Table 2). The median lethal concentrations (LC_50_) were 0.09, 0.15, and 0.21 mg/ml for CSd, CSF and CSO, respectively (Table 3). The old sample of *C. siliqua* had the highest mortality rate of 100% for larvae after 48 h of PT, whether it was in methanol extracts for all samples. The dry stem extract at 0.2 mg/ml had rates of 100% mortality (Table 3).

**Table 2:** Efficacy of *Ceratonia siliqua* extracts on *Culex pipiens* larval mortality, 24 and 48 h post-treatment.

Time (hr)	Treatment	Concentration (mg/ml)							
		0	0.01	0.025	0.05	0.1	0.2	0.5	1.0
24	CSF	0 ± 0^aH^	1.3 ± 1.33^aG^	5.3 ± 1.33^aF^	12.0 ± 2.31^bE^	26.6 ± 1.33^bD^	64.0 ± 2.31^bC^	89.3 ± 5.33^bB^	100.0 ± 0.00^aA^
	CSd	0 ± 0^aH^	2.6 ± 1.33^aG^	6.6 ± 1.33^aF^	14.6 ± 1.33^aE^	33.3 ± 1.33^aD^	70.6 ± 3.53^aC^	94.6 ± 1.33^aB^	100.0 ± 0.00^aA^
	CSO	0 ± 0^aH^	1.3 ± 1.33^aG^	2.6 ± 1.33^bF^	9.3 ± 1.33^cE^	18.6 ± 1.33^cD^	45.3 ± 2.67^cC^	80.0 ± 4.62^cB^	96.0 ± 2.31^bA^
	Temphos	91.1 ± 1.33							
48	CSF	0 ± 0^aH^	4.0 ± 0.00^abF^	8.0 ± 2.31^bE^	20.0 ± 0.00^aD^	41.3 ± 1.33^bC^	85.3 ± 2.67^bB^	100.0 ± 0.00^aA^	100.0 ± 0.00^aA^
	CSd	0 ± 0^aH^	5.3 ± 1.33^aF^	10.6 ± 1.33^aE^	20.0 ± 0.00^aD^	52.0 ± 2.31^aC^	100.0 ± 0.00^aA^	100.0 ± 0.00^aA^	100.0 ± 0.00^aA^
	CSO	0 ± 0^aH^	2.6 ± 1.33^bG^	4.0 ± 2.31^cF^	13.3 ± 1.33^bE^	33.3 ± 1.33^cD^	70.6 ± 2.67^cC^	90.6 ± 1.33^bB^	100.0 ± 0.00^aA^
	Temphos	100.0 ± 0.00							

a, b & c: There is no significant difference (*P* > 0.05) between any two means for each time, within the same column have the same superscript letter; A, B & C: There is no significant difference (*P* > 0.05) between any two means, within the same row have the same superscript letter. CSF: *Ceratonia siliqua* fresh stem extract, CSd: *Ceratonia siliqua* dried stem extract and CSO: *Ceratonia siliqua* old (stored) stem extract. Positive control: Temphos (1 mg/L).

**Table 3:** Lethal concentrations (ppm) of *Ceratonia siliqua* extracts on *Culex pipiens* larval mortality, 24 and 48 h post-treatment.

Time (hr)	Treatment	LC_50_ (Low-Up.)	LC_90_ (Low-Up.)	LC_95_ (Low-Up.)	Slope ±SE	Chi-squire (sig.)
24	CSF	0.15 (0.13–0.17)	0.51 (0.42–0.64)	0.72 (0.58–0.94)	2.386 ± 0.150	10.758 (0.056)
	CSd	0.09 (0.06–0.15)	0.32 (0.28–0.90)	0.46 (0.42–1.54)	2.333 ± 0.160	28.053 (0.000)
	CSO	0.21 (0.18–0.24)	0.81 (0.65–1.04)	1.18 (0.82–1.60)	2.198 ± 0.141	10.665 (0.058)
48	CSF	0.09 (0.06–0.14)	0.29 (0.23–0.59)	0.40 (0.33–0.90)	2.568 ± 0.167	29.201 (0.000)
	CSd	0.07 (0.04–0.11)	0.25 (0.20–0.48)	0.33 (0.28–0.77)	2.536 ± 0.166	30.870 (0.000)
	CSO	0.13 (0.10–0.18)	0.4 (0.35–0.77)	0.65 (0.49–1.18)	2.370 ± 0.149	17.449 (0.003)

CSF: *Ceratonia siliqua* fresh stem extract, CSd: *Ceratonia siliqua* dried stem extract and CSO: *Ceratonia siliqua* old (stored) stem extract.

#### Housefly larvicidal activity

All evaluated plant extracts exhibited markedly elevated death rates compared to the controls. After being treated with a plant extract at a high concentration of 25 mg/ml, the mortality rates of larvae were 100, 100 and 96% for CSF, CSd, and CSO, respectively. This was in contrast to the control groups, which had a mortality rate of 0% 24 h after treatment (Table 4). After 48 h of exposure, the fresh extract of *Ceratonia siliqua* L. had the highest mortality rate (100%) among the three treatments at 10 mg/ml concentration. The LC_50_ values for *C. siliqua* were 2.30, 2.84 and 3.86 mg/ml at 24 h and 1.80, 2.27 and 2.93 mg/ml at 48 h post-treatment for CSF, CSd, and CSO (Table 5).

**Table 4:** Efficacy of *Ceratonia siliqua* extracts on housefly larval mortality, 24 and 48 h post-treatment.

Time (hr)	Treatment	Concentration (mg/ml)						
		0	0.5	1.0	2.5	5.0	10.0	25.0
24	CSF	0 ± 0^aF^	8.0 ± 2.31^aE^	18.6 ± 2.67^aD^	42.6 ± 3.53^aC^	78.6 ± 2.67^aB^	97.3 ± 1.33^aA^	100.0 ± 0.00^aA^
	CSd	0 ± 0^aG^	6.6 ± 1.33^abF^	16.0 ± 2.31^bE^	36.0 ± 2.31^bD^	73.3 ± 1.33^bC^	89.3 ± 2.67^bB^	100.0 ± 0.00^aA^
	CSO	0 ± 0^aG^	5.3 ± 1.33^bF^	12.0 ± 2.31^cE^	28.0 ± 2.31^cD^	62.6 ± 1.33^cC^	80.0 ± 2.31^cB^	96.0 ± 2.31^bA^
48	CSF	0 ± 0^aF^	13.3 ± 1.33^aE^	24.0 ± 2.31^aD^	50.6 ± 2.67^aC^	88.0 ± 4.62^aB^	100.0 ± 0.00^aA^	100.0 ± .00^aA^
	CSd	0 ± 0^aF^	9.3 ± 1.33^bE^	18.6 ± 1.33^bD^	42.6 ± 1.33^bC^	82.6 ± 1.33^bB^	96.0 ± 1.33^bA^	100.0 ± 0.00^aA^
	CSO	0 ± 0^aG^	6.6 ± 1.33^cF^	13.3 ± 1.33^cE^	36.0 ± 2.31^cD^	77.3 ± 1.33^cC^	90.6 ± 1.33^cB^	100.0 ± 0.00^aA^

a, b & c: There is no significant difference (*P* > 0.05) between any two means for each time, within the same column have the same superscript letter; A, B & C: There is no significant difference (*P* > 0.05) between any two means, within the same row have the same superscript letter. CSF: *Ceratonia siliqua* fresh stem extract, CSd: *Ceratonia siliqua* dried stem extract and CSO: *Ceratonia siliqua* old (stored) stem extract.

**Table 5:** Lethal concentrations (ppm) of *Ceratonia siliqua* extracts on housefly larval mortality, 24 and 48 h post-treatment.

Time (hr)	Treatment	LC_50_ (Low-Up.)	LC_90_ (Low-Up.)	LC_95_ (Low-Up.)	Slope ±SE	Chi-squire (sig.)
24	CSF	2.32 (2.08–2.67)	7.84 (6.65–9.63)	11.03 (19.10-14.08)	2.452 ± 0.154	7.458 (0.113)
	CSd	2.84 (2.49–3.24)	10.33 (8.55–13.05)	14.90 (11.92–19.70)	2.285 ± 0.152	6.397 (0.171)
	CSO	3.86 (3.36–4.43)	16.59 (13.48–21.37)	25.08 (19.64–33.96)	2.023 ± 0.127	3.608 (0.461)
48	CSF	1.80 (1.26–2.60)	6.04 (4.62–10.96)	8.50 (6.51–16.91)	2.472 ± 0.170	14.111 (0.006)
	CSd	2.27 (2.00-2.58)	7.61 (6.46–9.25)	10.72 (8.85–13.54)	2.440 ± 0.150	9.370 (0.052)
	CSO	2.93 (2.56–3.35)	10.66 (8.62–13.99)	15.38 (11.96–21.33)	2.282 ± 0.170	5.168 (0.159)

CSF: *Ceratonia siliqua* fresh stem extract, CSd: *Ceratonia siliqua* dried stem extract and CSO: *Ceratonia siliqua* old (stored) stem extract.

**Fig. 5:**
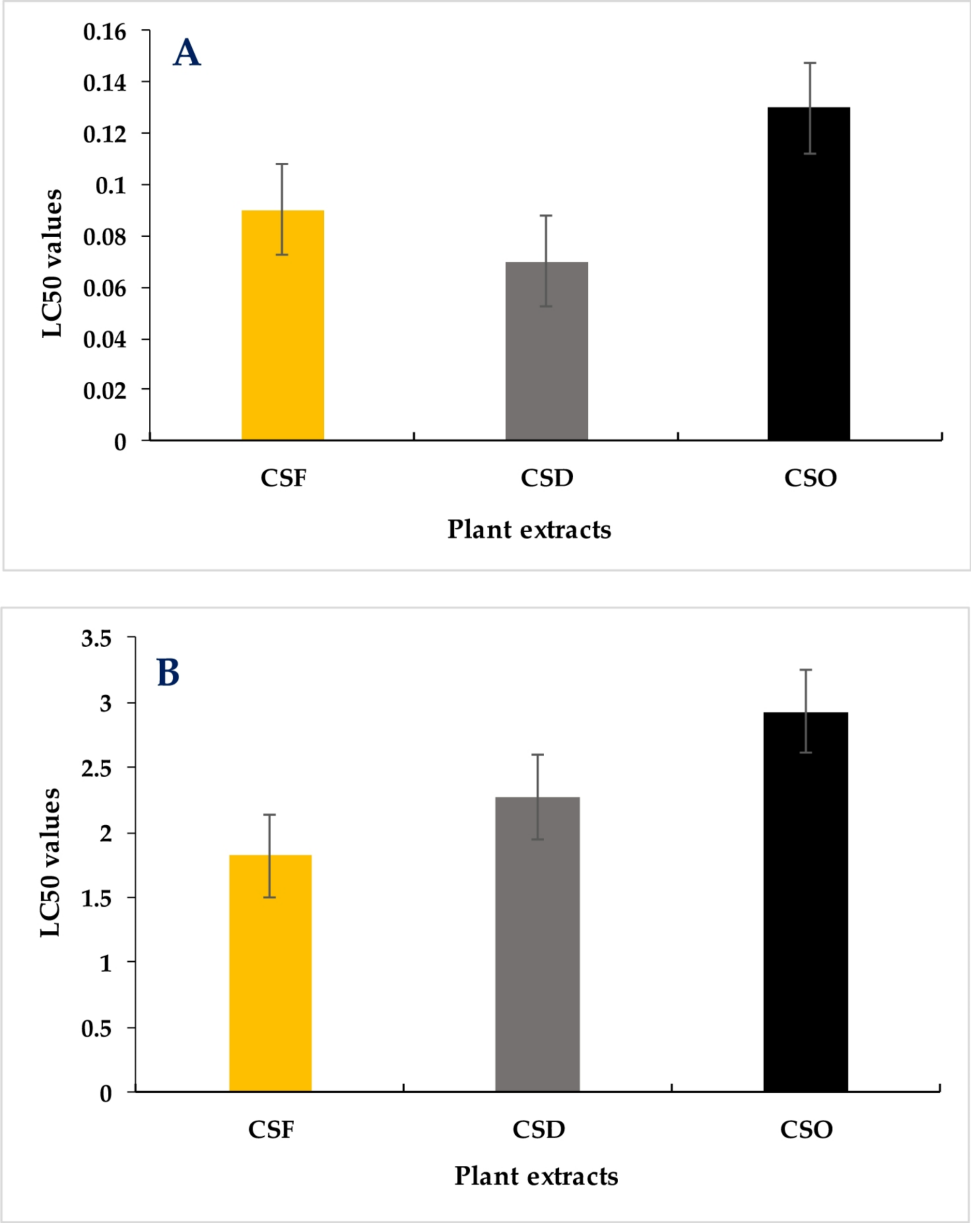
The mean number of larval mortalities induced by the effects of *Ceratonia siliqua* extracts against *Culex pipiens* (**A**) and *Musca domestica* (**B**), 48 h post-exposure.

The data indicated that CSD exhibited more toxicity to *Cx. pipiens* larvae compared to other plant extracts (Fig. 5a) and on other hand it was found that *C. siliqua* (CSF) more effective than other *Ceratonia siliqua* CSD and CSO based on LC_50_ (Fig. 5b).

## Discussion

Many of the important natural compounds found in plant extracts can be safely employed to control diseases and pests because of their natural degradation. Despite their advantages, biopesticides only account for 5% of the market for pesticides. However, with an average yearly growth rate of 9–20%, biopesticides are expanding quickly these days and are expected to surpass chemical pesticides in growth^60^. The use of chemical pesticides causes insects to become resistant, destroys helpful microbes and increases lingering issues that may endanger human health and the environment. Therefore, in order to manage pests, an environmentally friendly plant-based insecticide must be developed^61^.

Carob (*Ceratonia siliqua* L.) corresponds to an evergreen leguminous tree (Fabaceae family). The high phenolic content of numerous parts of carob has been deeply associated with several nutritional and functional benefits. Carob fruit and seeds are usually the edible part of the tree however the stems are regarded as agricultural waste. This study highlights the importance of reusing such waste for the search for potential phyoconstituents with environmental and medical importance.

Herein, the stems of Carob were collected along two successive seasons (old and fresh) and during the new season part of the collected stems was dried. Thus, three different extracts were prepared as the old (stored) stem extract CSO (2022–2023 season), the fresh stem extract CSF (2023–2024 season) and the dried stem extract CSd (2023–2024 season). The three extracts were compared phytochemically as detailed in the results section. The extracts were found rich in flavonoids, phenolic acids, tannins, fatty acids and others which came in line with the reported metabolites from literature.

CSd showed the highest number of identified components followed by CSO and CSF. The % identification was nearly equal in the negative ion mode for the three extracts while for the positive ion mode it followed the order of CSF > CSd > CSO. The differences in the localization of certain phytoconstituents or their relative percentages between the three extracts can be attributed to the difference between seasons in the temperature, degree of humidity, sunlight, drought, climatic changes. Besides, the differences revealed between the two extracts prepared within the same season (CSF and CSd) may be explained by the differences occurring upon drying of the plant which may activate or deactivate certain plant enzymes or metabolic pathways that may eventually lead to differences in the metabolic buildup of each of them^62–64^.

Upon reviewing literature on genus *Ceratonia*, many other studies showed interesting phytochemical components and biological activities that were worth mentioning as detailed below. Using HPLC-DAD ESI-Q-TOF-MS/MS, the hydro-methanol extracts (80/20 v/v) of *C. siliqua* pods and seeds were examined. 53 secondary bioactive metabolites from several classes (flavonoids, phenolic acids, tannins and non-phenolic chemicals) were detected in the hydro-methanol extracts of the pods and seeds. The antioxidant activity was demonstrated in ABTS (198.50 mmol Eq Trolox/100 g for pods and 201.04 mmol Eq Trolox/100 g for seeds), DPPH (22.24 mg/ml for pods and 26.37 mg/ml for seeds) and FRAP (0.39 mmol Eq Trolox/100 g for pods and 0.53 mmol Eq Trolox/100 g for seeds). Furthermore, the antioxidant activity determined by the DPPH technique was found to have very significant (*p* ≤ 0.01) correlation coefficients with total phenols (*r* = 0.943), orthodiphenols (*r* = 0.996), flavonoids (*r* = 0.880) and flavonols (*r* = 0.982)^51^.

In a zebrafish model, the antioxidant potential of carob pod water extract (CPWE) against oxidative stress caused by deltamethrin (DM), a pesticide that is commonly used worldwide, was examined both *in vitro* and *in vivo*. Trolox, BHA, and BHT standard antioxidants were used in various techniques to assess the extracted material’s *in vitro* antioxidant capacity. For *in vivo* tests, larvae were treated with 1–10 and 100 ppm CPWE for 4 h at 72 h after 4hpf zebrafish embryos were exposed to 10 ppb and 25 ppb DM for 120 h. It has been found that zebrafish exposed to DM during the developmental stage have significant body deformities, a lower survival rate, smaller eyes, shorter bodies and less locomotor activity during the dark phase^32^.

Six phenolic compounds were detected in the ethyl acetate fraction of *C. siliqua* (EACs) HPLC fingerprint. According to the *in vivo* findings, oral CCl_4_ administration raised the levels of renal and hepatic markers (ALT, AST, ALP, LDH, c-GT, urea and creatinine) in the experimental animals’ serum. Additionally, it raised the indicators of oxidative stress, which led to a rise in lipid peroxidation and a corresponding drop in the levels of enzyme antioxidants (SOD, CAT and GPx) in the kidney and liver. Experimental rats were pre-treated with 250 mg/kg (BW) of EACs *via* intraperitoneal injection for eight days, which avoided CCl_4_-induced abnormalities in the levels of kidney and liver indicators^65^.

Mass spectrometry (MS)-based metabolomics was used to evaluate the effects of geographic origin, ontogenetic modifications and thermal processing on the metabolome of *C. siliqua* pods. As a result, 70 principal metabolites from fruits were identified, primarily consisting of organic, amino and carbohydrate acids. Ultra-high-performance liquid chromatography-electrospray ionization high resolution mass spectrometry (UHPLC-ESI-HR-MS) analysis of secondary bioactive metabolites identified 83 signals in total. Tannins and flavonoids were identified as the primary signals that most significantly contributed to the discriminating of *C. siliqua* specimens. PCA models generated from GC-MS or UHPLC-MS were found to be effective instruments for differentiating *C. siliqua* specimens^33^.

The *in vitro* inhibitory capacity of carob (*C. siliqua* L.) leaf and pod acetone and ethanol extracts against the pectinolytic Gram negative *Pectobacterium atrosepticum* (Pca, CFBP-5384) bacteria, which causes potato soft rot, was assessed. To investigate Pca pathogenicity, LC/MS and GC/MS were used to evaluate potato (*Solanum tuberosum*, var *nicola*) tuber rot tissues that were taken following a 5-day bacterial inoculation.

Following a 5-day inoculation with Pca in a dark, moist room, *trans*/*cis* N-feruloyl-putrescine was detected in potato tuber. The Pca soft rot infection boosted the production of glycoalkoloid (*α*-chaconine and *α*-solanine), but this did not determine resistance. Numerous secondary metabolites were found, such as the fatty acids and phytoalexins solavetivone that are involved in plant defense responses. When combined with infected potato tuber extract (Pdt-Pca extract), acetone extract of carob leaf (FCA) demonstrated a synergistic antibacterial action against Pca and the greatest inhibitory effect (IC_50_ ¼ 1.5 mg/ml)^66^.

Nutritious proteins, lipids and phenolics can be found in the seed germ of European carob (*C. siliqua*) and South American algarrobo (*Prosopis* species). We analyzed and semi quantified flavonoids from the germ of three Argentinean algarrobo (*Prosopis alba*, *Prosopis nigra* and *Prosopis ruscifolia*) and one European carob species using reversed phase-HPLC-diode array detector and nanoflow-HPLC coupled to tandem mass spectrometry (MS/MS). Given the utilization of seed germ flour (SGF) in food applications, the patterns of glycosylated flavonoids were very comparable to one another, suggesting their molecular functional similarity and validating the taxonomic parentage of the species. Apigenin 6,8-*C*-di-glycoside isomers, specifically *iso*-schaftoside and schaftoside, were the most abundant phenolic compounds, making up 3.22–5.18 and 0.41–0.72 mg/g SGF, respectively. Prosopis had a lesser abundance of additional glycosylated derivatives of (*iso*)schaftoside than *C. siliqua* germ, which had comparatively higher levels. Food preparations made with *Prosopis* spp. and *C. siliqua* SGF may help regulate how humans digest carbs because apigenin 6,8-*C*-*di*-glycosides have been shown to be strong α-glucosidase inhibitors^52^.

HPLC/MS was used to evaluate the methanol extract of Egyptian *C. siliqua* and 36 chemicals were discovered in a preliminary manner. The presence of flavonoids (75.4% of plant dry weight) was primarily represented by two methylapigenin-*O*-pentoside isomers (20.9 and 13.7% of plant dry weight), with 26 compounds found in the negative mode accounting for 85.4% of plant dry weight and 10 compounds found in the positive mode representing 16.1% of plant dry weight. The discovery of the numerous compounds found in carob pods leads to a better knowledge of the varied health benefits that carob and its derivatives offer^28^.

Polyphenols are the primary chemicals of interest in carob leaves, which are the plant’s least studied component. The potential of advanced extraction methods including pressurized liquid (PLE), microwave-assisted (MAE) and ultrasound-assisted (UAE) extraction to extract these chemicals has not been fully investigated. Thus, this paper’s objectives were to optimize the temperature and time of carob leaf polyphenols’ PLE, MAE and UAE, characterize each compound using ultra-high-performance liquid chromatography tandem mass spectrometry (UPLCMS2) and assess the extracts’ antioxidant capacity. For PLE, 160 ◦C/5 min was the ideal temperature and duration, yielding a total phenolic content of 68.21 mg of gallic acid equivalents (GAE) g − 1. The ideal conditions for MAE and UAE were 70 ◦C/10 min, which resulted in total phenolic concentrations of 78.80 and 55.98 mg GAE g − 1, respectively. Extracts from all three extraction methods contained a total of 26 components, primarily myricetin, quercetin-3-rhamnoside and gallic acid. Carob leaf extracts obtained through advanced extraction have a high potential for use in functional food products, as evidenced by the high antioxidant activity (0.46–1.05 and 0.50–0.58 mmol TE g − 1 for FRAP and DPPH, respectively), which correlated with the polyphenolic content and was influenced by the ascorbic acid concentration (0.03–0.52 mg mL − 1)^67^.

The ethanol extract of *C. siliqua* leaves (CSEE) was tested for its cytotoxic, antibacterial and antioxidant qualities. The main components of the CSEE extract were determined to be flavonoids and phenolic acids by HPLC-DAD analysis. According to the DPPH test results, the extract had a strong scavenging ability with an IC_50_ of 302.78 _ 7.55 µg/mL, which was similar to that of ascorbic acid, which had an IC_50_ of 260.24 _ 6.45 µg/mL. An IC_50_ of 352.06 _ 12.16 µg/mL was also shown in the *β*-carotene test, indicating the extract’s capacity to prevent oxidative damage. The TAC assay showed an IC_50_ value of 165 _ 7.66 µg AAE/mg, whereas the ABTS assay showed IC_50_ values of 48.13 _ 3.66 TE µmol/mL, suggesting a considerable ability of CSEE to scavenge ABTS radicals^68^.

This study evaluated the efficacy of *C. siliqua*, carob plant extract against two insect species associated with medically relevant disorders and analyzed the extract’s antimicrobial properties. All *C. siliqua* extracts used in this study showed significant insecticidal effectiveness against mosquito and house fly larvae. The toxicity of the plant extracts, especially the newer (fresh and dry) extracts, exceeded that of the old stem extract. The methanolic extract of *C. siliqua* had a fatal impact on mosquito and house fly larvae, resulting in a 100% mortality rate among all treated larvae within 24 h. The study revealed that the larval mortality rate in both species increased with time, concentration and the solvent employed.

As mentioned, the carob tree, *C. siliqua*, is currently considered one of the most valuable fruit and forest trees in various fields and sectors, especially pharmaceutical activities. Its importance has increased significantly in recent years due to its many compounds, including polyphenols, flavonoids, carbohydrates, minerals and proteins. This has led the carob tree to show antihypertensive, antihyperglycemic, anti-obesity, antidepressant, antidiarrheal, antioxidant and other activities. Although this plant compound has demonstrated clear activity against numerous microbes^69,70^, its use as a biocide against insects or parasites appears to be new.

Plant extracts and essential oils still control many medical, veterinary and agricultural pests. Environmentally friendly plant extracts and essential oils are particularly effective in killing mosquito larvae and insects in general. Mostafa, et al.^71^ conducted a study that uncovered plants as a rich source of biological materials, containing a diverse array of potential phytochemicals that target specific targets. The leaf extracts of *Delonix regia*,* Bougainvillea glabra*,* Platycladus orientalis* and *Lantana camara* were very good at killing insects and microbes and protecting cells from damage. They demonstrated that acetone extracts were particularly harmful to *Cx. pipiens* (99.0–100%, 72 h after treatment), with the LC_50_ values for, *P. orientalis*,* L. camara*,* B. glabra* and *D. regia* being 71.1, 95.4, 142.8, 189.5 ppm, respectively.

Mohamed, et al.^72^ assessed the efficacy of extracts from *Melia azedarach*,* Nerium oleander*,* Ricinus communis*,* Lantana camara* and *Withania somnifera* against *Cx. pipiens* larvae. Methanol extracts exhibited greater toxicity against *Cx. pipiens* (95–100%, 24 h post-treatment) compared to aqueous extracts (63–91%, 24 h post-treatment). Using methanol extracts of oleander (LC_50_ = 158.92 ppm) and castor bean (LC_50_ = 175.04 ppm) to kill mosquito larvae 24 h after treatment has been very effective. Larvicidal and adulticidal effects against *Cx. pipiens* were demonstrated in vitro, supported by field evaluations using essential oils such as fennel and tea oil and their nanoformulations. Biological evaluation of the nanoformulations and essential oils showed promising larvicidal and adulticidal activity^73^.

Essential oils demonstrated high to moderate toxicity towards house fly larvae. Tests revealed that a 10% concentration of *Rosmarinus officinalis*,* Cinnamomum verum*,* Cyperus rotundus*,* Melaleuca alternifolia*,* Piper nigrum* and *Aloe vera* essential oils (EOs) completely killed third-instar larvae fed on treated breeding medium. However, the same concentration of EOs also caused the death of 91–100% of the larvae fed on contact-treated filter paper. Essential oils *of R. officinalis* and *C. verum* effectively eliminate housefly larvae, extend larval and pupal growth and boost the inhibition rate to 100%^74^.

Compounds in plants, such as flavonoids, alkaloids, esters, glycosides and fatty acids, can kill insects in a number of ways, including repelling or attracting them, preventing them from feeding, poisoning them, slowing their growth, or killing them chemically^26,75^. Worldwide, people use tea leaves, *Camellia sinensis*, for their psychoactive properties and health benefits. Young tea leaves contain levels of catechins (catechin, gallocatechin and catechin gallate), methylxanthines (caffeine and theophylline), flavonoids, vitamins, proteins and glycosides (kaempferol and myricetin). In addition, catechins have antiviral, antibacterial, antimalarial, anticancer, antioxidant, anti-inflammatory, anti-aging, anti-arthritic and anti-insect properties^76,77^.

Many plants, both short-lived (herbaceous) and long-lived (perennial), have useful chemicals that are effective in herbal medicine and other medical areas. Some of these plants, like *Mentha arvensis*,* Rosmarinus officinalis*,* Eucalyptus camaldulensis*, and *Cyperus rotundus*, are also good at killing mosquito larvae and other insects^78,79^.

Numerous studies have demonstrated that plant extracts, methanol, or acetone are more effective in exterminating mosquito larvae or door larvae compared to other solvents^80^. These results align with Bosly^81^, which ranked the extremely hazardous leaf extracts in the following order of toxicity to mosquito larvae: acetone, methanol, aqueous and hexane. The choice of solvent for extracting phytochemicals may influence the efficacy of plant extracts against specific mosquito species. A similar study looked at how ethanolic extracts from the leaves and fruits of *Physalis angulata* L. killed *Anopheles* mosquito larvae. The leaf extracts at concentrations of 5%, 10%, 15% and 20% resulted in 61%, 80%, 91% and 92% mortality, but the fruit extracts at identical concentrations produced 38%, 47%, 72% and 83% mortality, respectively. A mixture of leaf and fruit extracts had synergistic effects, resulting in mortality rates of 67%, 84%, 91% and 95% at identical doses and durations^82^.

In addition to using plant extracts and essential oils to repel insect pests, a contemporary trend involves using plant waste as an effective biocide against various pests. The work of Mohamed, et al.^74^ indicate that there is scant information regarding the conversion of green waste into biocides. This study examines the feasibility of using green waste as a novel biopesticide against *Cx. pipiens* mosquito larvae^83,84^. The present investigation revealed that plant extracts from *Punica granatum* (98.4% mortality), *Citrus sinensis* (92% mortality), *Brassica oleracea* (88%), *Oryza sativa* (81.6%) and *Colocasia esculenta* (53.6%) were highly effective in eliminating *Cx. pipiens* larvae 24 h post-treatment. The LC_50_ values were 314.43, 370.72, 465.59, 666.67 and 1798.03 ppm for *P. granatum*,* C. sinensis*,* B. oleracea*,* O. sativa* and *C. esculenta*, respectively.

This study looked at the possible trade-offs that might come up when using plant extracts that kill pests on legume crop yields and the ecological services that natural pest enemies provide. The efficacy of six recognized pesticidal plants (*Bidens pilosa*,* Lippia javanica*,* Tephrosia vogelii*,* Tithonia diversifolia*,* Lantana camara and Vernonia amygdalina*) was evaluated against positive and negative controls regarding their effects on the yields of bean (*Phaseolus vulgaris*), cowpea (*Vigna unguiculata*) and pigeon pea (*Cajanus cajan*) crops, as well as the population dynamics of significant indicator pest and predatory arthropod species. Analysis of field trials showed that treatments for pesticidal plants often had crop results that were similar to those seen with the synthetic pesticide lambda-cyhalothrin. The most effective plant species were *T. vogelii*,* T. diversifolia* and *L. javanica*. This study shows that using extracts from plants that are good at killing pests can be just as effective as synthetic insecticides at protecting crops. At the same time, it can reduce the negative effects on three-trophic systems and protect non-target arthropods that provide important ecosystem services like pollination and pest control^85^.

## Conclusion

In conclusion, this comparative phytochemical study was performed on three *Ceratonia siliqua* L. stem samples from two fruiting seasons. The UPLC/MS for the three samples leads to the tentative identification of fifty-four secondary metabolites from different phytochemical classes. The dried stem extract (CSd) exhibited higher number of components compared to the other samples. The % identification showed no significant difference in the negative ion mode while in the positive ion mode the CSF sample had higher percentage compared to CSd and CSO for the three extracts while for the positive ion mode it followed the order of CSF > CSd > CSO. The variation in the phytochemical composition between the three samples may be due to the effect of drying and season of collection. Plant methanol extracts demonstrated significant insecticidal activity against mosquito larvae, *Cx. pipiens*, and housefly larvae, *M. domestica*. The CSd was the most effective on *Cx. pipiens* larvae with 1.0 mg/mL, while CSF was the most beneficial on *M. domestica* larvae 24 and 48 h after treatment with a 25 mg/mL concentration. We concluded that the most effective extracts for killing mosquito and house fly larvae were CSd and CSF, followed by the CSO extract. *C. siliqua* extracts may serve as an effective agent for specific vector-borne infection control.

## Data Availability

The datasets used and/or analyzed during the current study are available from the corresponding author upon reasonable request.
